# An unusual polypoid lesion in the cecum

**DOI:** 10.1002/ccr3.2916

**Published:** 2020-05-04

**Authors:** Faidon‐Marios Laskaratos, Hanan El‐Mileik

**Affiliations:** ^1^ Gastroenterology Department University College London Hospitals NHS Foundation Trust London UK; ^2^ Gastroenterology Department Queen’s Hospital Barking Havering and Redbridge University Hospitals NHS Trust Romford UK; ^3^ Endoscopy Unit Chartwell Private Hospital Leigh‐on‐Sea UK

**Keywords:** appendix, cecum, colonoscopy, lesion

## Abstract

The inverted appendix or appendiceal stump is a rare finding that may be identified at colonoscopy. This may cause diagnostic confusion and be misinterpreted as a polyp with a risk of peritonitis if excised endoscopically.

## Graphical abstract

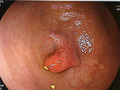

## 1 REPORT

We describe a rare endoscopic finding of an inverted appendiceal stump identified at a diagnostic colonoscopy. This may cause diagnostic confusion and be misinterpreted as a polyp with a risk of peritonitis if excised endoscopically. Therefore, gastroenterology physicians should be aware of this possible finding.

**FIGURE 1:**
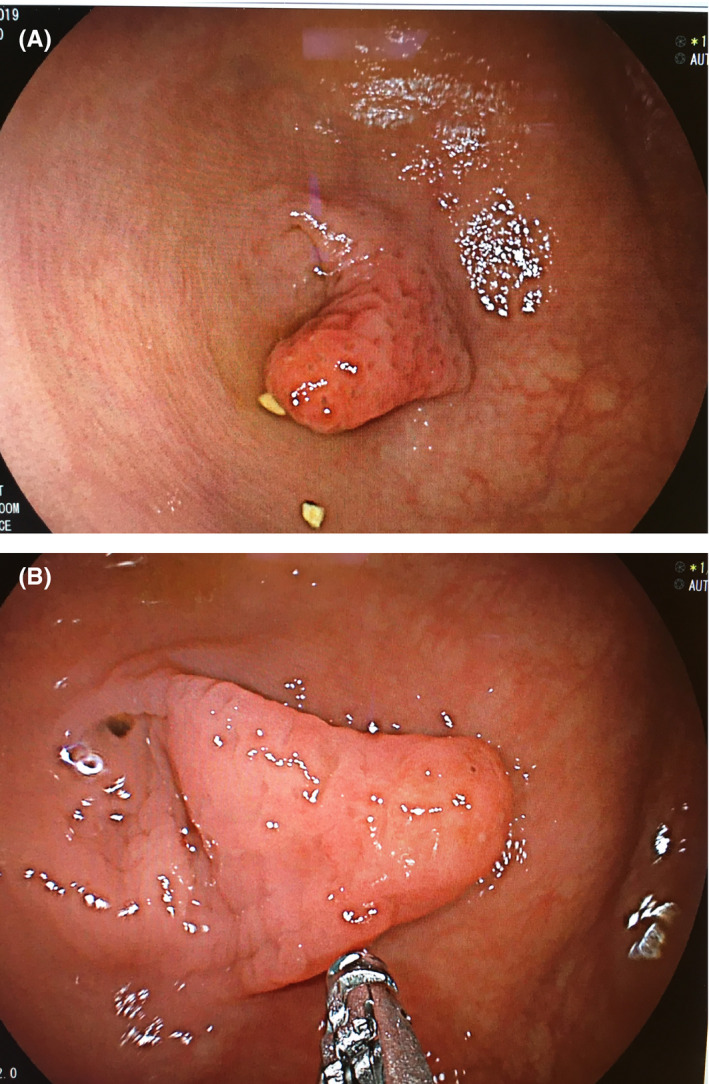
Endoscopic view of a polypoid lesion arising from the appendiceal orifice, consistent with an inverted appendiceal stump

A 50‐year‐old male with a past medical history of a previous appendicectomy in childhood was referred for a colonoscopy to investigate symptoms of abdominal pain and bloating, as well as occasional episodes of rectal bleeding. The colonoscopy revealed an elongated polypoid lesion in the cecum at the location of the appendiceal orifice, consistent with an inverted appendiceal stump (Figure 1A,B). Biopsies were taken which showed large bowel mucosa with mild chronic inflammation.

The inverted appendix is a rare, benign endoscopic finding with an incidence of 0.01%.^1^ It may be asymptomatic or be associated with symptoms, such as abdominal pain, vomiting, or rectal bleeding.^2^ This endoscopic finding can often cause diagnostic confusion, as it may be mistaken for a cecal polyp and removed at colonoscopy, which carries a high risk of subsequent peritonitis.^2^ The location of the lesion in relation to the appendiceal orifice and its normal mucosal surface (pit pattern) should alert the endoscopist as to the true nature of this finding.^2^ Finally, it should be noted that a history of previous appendicectomy does not preclude this diagnosis, since after appendicectomy the residual appendiceal stump may be inverted into the cecum.^2^

## CONFLICT OF INTEREST

None declared.

## AUTHOR CONTRIBUTIONS

Both authors contributed equally to the preparation of this manuscript.
